# A Radiomics-Based Classifier for the Progression of Oropharyngeal Cancer Treated with Definitive Radiotherapy

**DOI:** 10.3390/cancers15143715

**Published:** 2023-07-22

**Authors:** Darwin A. Garcia, Elizabeth B. Jeans, Lindsay K. Morris, Satomi Shiraishi, Brady S. Laughlin, Yi Rong, Jean-Claude M. Rwigema, Robert L. Foote, Michael G. Herman, Jing Qian

**Affiliations:** 1 Department of Radiation Oncology, Mayo Clinic, Rochester, MN 55905, USA; 2 Mayo Clinic Graduate School of Biomedical Sciences, Mayo Clinic, Rochester, MN 55905, USA; 3 Department of Radiation Oncology, Mayo Clinic, Phoenix, AZ 85054, USA

**Keywords:** PET, radiomics, oropharyngeal cancer, definitive radiotherapy, progression

## Abstract

**Simple Summary:**

Oropharyngeal cancer is the most common type of head-and-neck squamous cell carcinoma. Although patients with HPV-associated cancers have a better prognosis than patients with HPV-negative cancers, there is a lack of robust biomarkers that describe the relative risk for disease progression. To investigate this problem, we extracted quantitative descriptors from medical images (known as radiomics features) that could not otherwise be assessed by the naked eye. We built a machine learning model based on clinical and radiomics features to predict whether a patient will exhibit disease progression at 2 years post-treatment. These findings are important for identifying patients treated with definitive radiotherapy with low and high risk of disease progression and formulating patient-specific treatment strategies.

**Abstract:**

In this study, we investigated whether radiomics features from pre-treatment positron emission tomography (PET) images could be used to predict disease progression in patients with HPV-positive oropharyngeal cancer treated with definitive proton or x-ray radiotherapy. Machine learning models were built using a dataset from Mayo Clinic, Rochester, Minnesota (n = 72) and tested on a dataset from Mayo Clinic, Phoenix, Arizona (n = 22). A total of 71 clinical and radiomics features were considered. The Mann–Whitney U test was used to identify the top 2 clinical and top 20 radiomics features that were significantly different between progression and progression-free patients. Two dimensionality reduction methods were used to define two feature sets (manually filtered or machine-driven). A forward feature selection scheme was conducted on each feature set to build models of increased complexity (number of input features from 1 to 6) and evaluate model robustness and overfitting. The machine-driven features had superior performance and were less prone to overfitting compared to the manually filtered features. The four-variable Gaussian Naïve Bayes model using the ‘Radiation Type’ clinical feature and three machine-driven features achieved a training accuracy of 79% and testing accuracy of 77%. These results demonstrate that radiomics features can provide risk stratification beyond HPV-status to formulate individualized treatment and follow-up strategies.

## 1. Introduction

Oropharyngeal cancer (OPC) is the most prevalent type of head-and-neck squamous cell carcinoma. Despite a decline in the incidence of head-and-neck-squamous cell cancers in recent decades [1], human papillomavirus (HPV) has driven an increase in the incidence of OPC across various demographic groups [2,3].

HPV-positive OPC is staged as a disease entity that is distinct from HPV-negative OPC, with favorable prognosis associated with HPV-positive cases [4,5]. Patients with HPV-positive OPC are younger at diagnosis, have fewer medical comorbidities, and have a higher survival rate compared to their HPV-negative counterparts [4]. The standard treatment for OPC involves a course of single daily fractionated radiation with or without concurrent chemotherapy, with cisplatin being the standard of care for advanced OPC [6,7]. Both approaches incur high rates of acute and late toxicities in a young population that could experience treatment sequalae for several decades [7]. With the objective of reducing such treatment-related toxicities, recent clinical trials have aimed to de-escalate treatment for HPV-positive patients while preserving high tumor control rates [8]. Clinical trials that evaluated a substitute for cisplatin in low-risk HPV-positive patients reported inferior overall survival and progression-free survival with no reduced toxicities [9,10]. Therefore, markers beyond HPV status are essential for distinguishing between low- and high-risk subgroups and predicting favorable or unfavorable responses.

In OPC patients, the majority of progressions occur during the first 2 years after treatment [11]. Current clinical practice for treatment follow-up is based on a patient’s risk of relapse. Generally, follow-up occurs every 6–12 weeks for the first year, every 3 months for the second year, and less frequently thereafter. Pre- and post-treatment 18-F-fluorodeoxyglucose positron emission tomography (PET)-computed tomography (CT) is routinely performed for OPC patients, and it plays a central role in the detection of locoregional and distant progression before patients present with symptoms. Anzi et al. reported lower mortality among patients with surveillance PET-CT imaging than patients without surveillance imaging [12]. Nevertheless, several challenges remain regarding surveillance PET-CT imaging: (i) follow-up PET-CTs can be difficult to interpret given persistent inflammatory changes that are often visualized post-treatment, (ii) post-treatment imaging remains understudied, with suboptimal schedules resulting in unnecessary scans too soon (or late) relative to when progression occurs, and (iii) the number of robust biomarkers for the early assessment of the risk of cancer progression is limited. The lack of optimal surveillance imaging paradigms and robust predictive markers of early and late local, regional, and distant progression present an unmet clinical need that must be addressed to provide guidance for personalized follow-up and treatment strategies.

Radiomics presents a new paradigm in medical imaging with a shift from visual interpretation to the extraction of quantitative descriptors, known as “features”, that cannot be reliably assessed through visual inspection [13]. Radiomics features consist of high-dimensional sets of mathematically defined quantities that can be analyzed computationally to provide diagnostic and prognostic information [14,15]. Previous studies have shown that features from pre-treatment PET images, such as standard uptake value, metabolic tumor volume, total lesion glycolysis, and textural features, provide significant prognostic information in OPC patients [16,17,18,19,20]. Folkert et al. investigated whether multivariable models that combine clinical and PET imaging features had better predictive power than the individual features alone [21]. In terms of local progression, the best model they reported consisted of metabolic tumor volume and a texture feature. The model retained predictive power (AUC = 0.68, sensitivity = 0.67, specificity = 0.70) when tested on an independent cohort. Vallieres et al. evaluated multivariable models using clinical, CT, and PET-CT radiomics features to predict locoregional and distant disease progression in various head-and-neck cancers [22]. For both outcomes, they demonstrated that radiomics features have increased predictive performance when combined with clinical variables. They reported a model that combined three PET-CT radiomics variables and four clinical variables as providing the best performance (accuracy = 0.67, sensitivity = 0.63, specificity = 0.68) for locoregional progression on an independent testing cohort. While several studies have built encouraging radiomics-based predictive models, the use of single-scanner and/or single-institution data, as well as the lack of stratification for HPV status, has been a key limitation for the generalizability of these predictive models.

We present a study on a cohort of HPV-positive patients who underwent pre-treatment PET-CT on various scanners and received definitive radiation with or without chemotherapy at our institution. In this context, we hypothesize that radiomics features could be used as risk-stratifying factors to guide individualized treatment and follow-up. We built multivariable machine learning models using clinical and radiomics features to predict disease progression at two years post-treatment and performed testing on an external dataset from a different institution. In the modern era, this provides crucial prognostic information to aid in defining low- and high-risk subgroups for cancer progression after definitive radiotherapy.

## 2. Materials and Methods

### 2.1. Patient Cohorts

A retrospective chart review was performed of patients diagnosed with OPC who received definitive external beam radiotherapy (protons or x-rays) with or without chemotherapy. These patients were treated at Mayo Clinic in Rochester, Minnesota (MCR), between May 2013 and April 2020 and Mayo Clinic in Phoenix, Arizona (MCA), between August 2013 and December 2020. Inclusion criteria were laboratory-confirmed HPV-positive carcinoma and availability of pre-treatment PET-CTs and radiotherapy treatment planning CTs. Additionally, patients were included if the period between the end of treatment and the chart review was at least 2 years or if they had developed local, regional, or distant disease progression. Patients were excluded if they had previously received radiotherapy or surgery. The Institutional Review Board at Mayo Clinic approved this study.

The clinical characteristics of the patient cohorts are summarized in Table 1. The MCR and MCA cohorts were composed of 72 patients and 22 patients, respectively. The pre-treatment PET-CT scans were performed using standard clinical procedures; given the retrospective nature of this work, there were no specific technical requirements for inclusion of image acquisition protocols.

The MCR dataset was used for feature engineering, model building, and model validation. The MCA group was not exposed to any feature engineering, model building, or model validation; rather, it was kept as an independent external cohort to test the performance and generalizability of the final models.

### 2.2. Feature Extraction

Patient demographics and clinical characteristics were collected, including age at diagnosis, primary disease site, type of radiotherapy, and type of chemotherapy. Pathological characteristics that classify tumor, node, and metastases were scored according to the 8th Edition of the American Joint Committee on Cancer (AJCC) staging system and treated as three distinct features represented only by their respective numerical descriptor. This was done since several of the data processing techniques employed are more appropriate for numerical data than categorical data. Follow-up data, including local, regional, and distant progression, as well as time to progression, were recorded. We defined the binary outcome for any type of progression (progression/progression-free) as the label for machine learning modeling. Other clinical features, such as smoking status, number of pack years, and number of chemotherapy cycles, were not included because they were not consistently reported in the patient electronic medical record. These features were removed, rather than excluding individual patients, to maintain statistical power.

MIM software (version 7.2.7, MIM software Inc., Cleveland, OH, USA) was used to develop a semi-automatic segmentation workflow based on standard uptake value (SUV) thresholding. The thresholding was employed to reduce any deviations caused by SUV variability between patients. Two volumes of interest were generated for each patient: regions of interest (ROIs) and largest ROI. For each patient, we defined the normal tissue SUV using a volumetric SUV mean of a 5 cm sphere centered in the liver. The ROI volume was delineated quantitatively using a tumor-to-normal tissue SUV ratio of 1.5. The largest ROI volume consists of the largest connected component of the ROI volume. To visually confirm that the SUV-based segmentation was consistent with the anatomical tumor location, we used rigid registration to transfer the physician-generated tumor contours from the planning CT to the pre-treatment PET image and ensured that they overlapped with the location of the ROIs and largest ROI. The volumes were resampled to 2 × 2 × 2 mm^3^ to create isotropic voxels.

**Figure 1:**
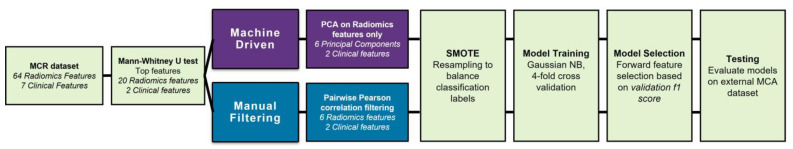
Schematic workflow for feature extraction, dimensionality reduction, data balancing, model training, and testing.

We extracted radiomics features using PyRadiomics, the open-source Python package, which is also involved in the efforts by the Image Biomarker Standardization Initiative team [23]. As in previous work [24,25], our in-house software incorporated Python’s scikit-learn packages [26] to build a pipeline for the processing and extraction of features. For each volume of interest, we calculated 14 shape-based features and 18 intensity-based features. A total of 64 radiomics features were calculated. Because our dataset consists of imaging data from different scanners and institutions, we did not include texture-based features in our analysis. Texture-based features are highly influenced by imaging acquisition parameters and the noise profile of the scanner; therefore, heterogeneity in the source of the imaging data may introduce uncertainty on the generalizability of these second-order features. The schematic in Figure 1 illustrates the techniques sequentially employed for feature selection, dimensionality reduction, data balancing, and machine learning modeling.

### 2.3. Dimensionality Reduction

All clinical and radiomics features were investigated for their predictive value relative to disease progression post-treatment in the MCR group. We used the Mann–Whitney U test to evaluate whether each feature was significantly different between patients with and without progression. The features were ranked from low to high based on their p-values. The top 2 clinical features and the top 20 radiomics features were selected for further analysis. In order to further reduce the number of variables, we investigated two dimensionality reduction methods and defined a set of features for each: i) a manually filtered set and ii) a machine-driven set.

The manual filtering process consisted of computing the bivariate Pearson correlation coefficient for each possible pair of the 22 features selected in the previous section. For every group of features with a correlation coefficient larger than 0.8, one feature in each group was selected based on explainability and all other features were excluded. In other words, we selected the radiomics features with the simplest mathematical definitions that enabled understanding of the tumor’s phenotype. The remaining set of 2 clinical and 6 radiomics features were normalized with a standard scaler by subtracting their mean and scaling to unit variance. The fitted scaler was saved, and the same scaler was recalled when the MCA data were tested. These 8 features were defined as the manually filtered set and used for further processing.

The machine-driven process involved the normalization and transformation of features. Each of the 22 features selected via the Mann–Whitney U test was normalized with a standard scaler by subtracting its mean and scaling to unit variance. Because a high correlation is expected for a given radiomics feature extracted from our two volumes of interest, principal component analysis (PCA) was performed on the 20 radiomics features to transform the correlated features into a smaller set of uncorrelated features (principal components) while preserving the majority of information in the dataset [26]. The generated principal components were ranked from high to low based on their explained variance, i.e., the amount of variability in the dataset explained by each component. The top 6 principal components with the largest explained variance were selected because they corresponded to the fewest number of components with an explained variance sum greater than 95%. The fitted scaler and PCA models were saved, and the same scaler and PCA models were recalled when the MCA data were tested. The machine-driven set, composed of the top 2 clinical features and the selected 6 principal components, was used for further processing.

### 2.4. Data Balancing

In this application, the utility of the eventual predictive model is based on its performance on the minority class, i.e., progression events. The incidence of any progression at two years post-treatment was 19.4% and 31.8% for the MCR and MCA cohorts, respectively. The imbalance between progression and progression-free events presents a challenge, in that machine learning models trained on imbalanced data may favor the class with the larger number of samples (progression-free events) and perform poorly in the prediction of progression events. To alleviate such a data imbalance problem in the MCR dataset, we adopted a combination of data over- and undersampling via SMOTETomek (The Synthetic Minority Over-Sampling Technique and Tomek), the full details of which are explained elsewhere [27]. Briefly, SMOTE creates new synthetic events by interpolating between existing progression events using k nearest neighbors [28]. Then, undersampling is performed by identifying Tomek links or pairs of events that lie closely in feature space but belong to opposite classes, and removing the event that belongs to the progression-free class [27]. The resulting dataset has the same number of progression and progression-free patients. For both the manual filtering and machine-driven methods, the implementation of SMOTETomek used the same random state seed as input. Data balancing was only performed on the MCR (training) dataset and not on the MCA (testing) dataset.

### 2.5. Feature Selection, Model Construction, and Testing

Several machine learning classifiers were considered due to their popularity and previous success, namely random forest, logistic regression, AdaBoost, and Gaussian Naïve Bayes (NB) [21,22,23,29]. Gaussian NB with default hyperparameters showed the most robust performance in terms of stability between training and cross-validation accuracy. Therefore, in this work we present results only from the Gaussian NB classifier. A NB classifier uses the training data to learn the conditional probability of each feature given the class label. It assumes that features are mutually independent and uses Bayes’ theorem to compute the probability of the classes given an instance of the features [30]. Gaussian NB assumes that the features within each class are drawn from a Gaussian distribution. For multivariable models, NB computes the product of all the probabilities from every feature and selects the class with the highest posterior probability.

Gaussian NB models were trained on the MCR dataset using each of the two sets of features, the manually filtered set and the machine-driven set. Because of the limited number of patients in the training MCR dataset and to reduce the chance of overfitting, we performed training using 4-fold cross-validation. We used a forward feature selection scheme to construct sets of models of increased complexity, i.e., number of input features from 1 to 6 features. The first step in our forward feature selection was to train all possible single-variable models and select the model with the best performance. Then, we trained incrementally more complex models by adding one feature at a time and retaining the feature that led to the best model performance. The performance metric that we used for model selection was the cross-validation f1 score, which represents the average f1 score across all 4 cross-validation trials. This approach facilitates the analysis of accumulated model learning, reveals when overfitting becomes a problem, and illustrates the complementary information provided by different features.

The 12 selected models (6 models from the manually filtered set and 6 models from the machine-driven set) were independently tested on the external MCA dataset. We evaluated prediction performance using accuracy of classification of binary endpoint (any progression at two years post-treatment).

## 3. Results

### 3.1. Dimensionality Reduction

**Figure 2:**
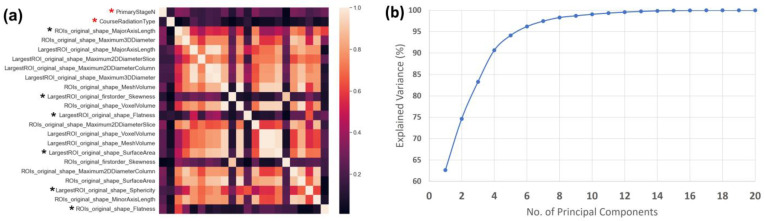
(**a**) Correlation matrix of 2 clinical and 20 radiomics features selected to be significantly different between progression and progression-free cohorts via the Mann–Whitney U test. The horizontal axis (columns) represents the same set of 22 features in the same order as presented in the vertical axis (rows). The manually filtered set is composed of 2 clinical features (red stars) and 6 radiomics features (black stars). (**b**) Explained variance as a function of the number of principal components obtained from PCA transformation of 20 radiomics features.

The 2 clinical features and 20 radiomics features identified as being different between progression and progression-free patients via the Mann–Whitney U test are listed in Figure 2a in the form of a correlation matrix. The manually filtered set that resulted from correlation filtering consists of the 2 clinical features and 6 radiomics features marked with an * in Figure 2a. The 20 radiomics features listed in Figure 2a were used to generate the principal components for building the machine-driven set. As shown in Figure 2b, the fewest number of principal components with an explained variance sum greater than 95% are the first 6 principal components, which make up the machine-driven set along with the 2 clinical features. Both of these dimensionality reduction methods reduced the number of features from 71 features to 8 features in each of the two sets defined.

### 3.2. Feature Selection, Model Construction, and Testing

All possible single-variable models were trained for the manually filtered features set and machine-driven features set. The ‘Radiation Type’ clinical feature, which codes radiation modality (x-rays or protons), was identified as the best single-variable model (based on the cross-validation f1 score) in both feature sets. For each feature set, models with 1 to 6 input features were sequentially built by adding one feature at a time and retaining the feature that resulted in the best cross-validation f1 score. Table 2 summarizes the features selected by each step of our forward feature selection scheme.

**Figure 3:**
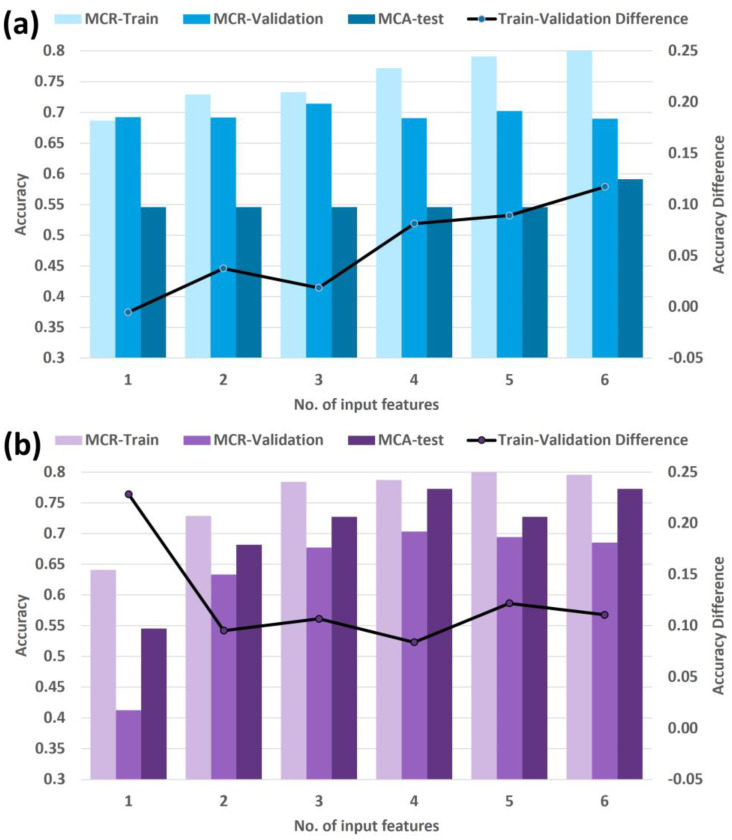
MCR training, MCR cross-validation, and MCA testing accuracy for models of different number of input features based on the (**a**) manually filtered and (**b**) machine-driven sets. Refer to Table 2 for the list of features used.

Figure 3 shows the MCR training, MCR cross-validation, and MCA testing accuracy for each selected model. Features from the manually filtered set maintained similar cross-validation accuracy with increasing number of input features (Figure 3a); likewise, features from the machine-driven set maintained similar cross-validation accuracy for models with 2 or more features (Figure 3b), which is an indication of the robustness of using the selected features in these two sets.

The models from the manually filtered set showed improved training accuracy as the number of features increased (Figure 3a), demonstrating that the models were incrementally learning each time a new feature was added. However, the increasing difference between training accuracy and cross-validation accuracy for models with three or more features is a possible indication of overfitting. The 3-feature model for the manually filtered set had the highest cross-validation accuracy (71%); however, with a training accuracy of 73% and a testing accuracy of 54% (sensitivity = 0.86, specificity = 0.40), overfitting was confirmed, hampering the generalizability of the model.

Similarly, the models from the machine-driven set showed incremental training accuracy up to the 5-feature model, with a slight decrease for the 6-feature model (Figure 3b). The difference between training accuracy and cross-validation accuracy decreased with the number of features with a minimum at the 4-feature model, then increasing for the 5- and 6-feature models. These trends suggest reasonable generalizability up to the 4-feature model and overfitting for the more complex models, which is supported by the rise and plateau in testing performance in Figure 3b. The 4-feature model for the machine-driven set had the highest cross-validation accuracy (70%), with training accuracy of 79% and testing accuracy of 77% (sensitivity = 0.86, specificity = 0.73).

## 4. Discussion

In this work, we demonstrated that a machine learning classifier based on pre-radiation clinical and PET radiomics features can be used to predict any progression of HPV-positive OPC patients at two years post-treatment. These findings are valuable for classifying patients as low- or high-risk and formulating individualized treatment and follow-up strategies after definitive radiotherapy.

This multi-institutional study consisted of a training set of MCR patients and a testing set of MCA patients. PET images were acquired following general clinical guidelines without requiring specific acquisition settings for inclusion. Therefore, this work represents a multi-scanner and multi-institutional dataset, rather than a homogeneous single-institution and/or single-scanner dataset that would limit the generalizability of radiomics-based models [14].

The SUV-based normalization and thresholding method used to define the volumes of interest appears to be an effective method to reduce interpatient SUV variability and promote generalizability between scanners and institutions. While we excluded texture features from our analysis due to their instability, this behavior is consistent with previous literature. Cheng et al. indicated that textural radiomics features may be influenced by imaging processing parameters and the noise profile of the scanners [31], and Folkert et al. found that texture features obtained from two different scanners showed significant predictive power only after a smoothing method was applied [21]. Future work could consider alleviating this challenge via data harmonization to reduce the scanner effect [32].

We compared two dimensionality reduction strategies to reduce the number of clinical and radiomics features used for modeling. Our forward feature selection approach facilitated the understanding of how the incremental number of input features improved learning, and it revealed when overfitting became a problem. On the one hand, the manually selected features are simple morphological and statistical definitions describing the tumor, but they exhibited overfitting for a lower number of input features compared to the machine-driven set. Their inferior performance in multivariable models indicates a lack of complementary information between the features. On the other hand, machine-driven features generated via PCA are more difficult to interpret, but our results demonstrate that they successfully combine the complementary value of different features to inform predictions. An interesting next step would be to study these principal components and how they map to the set of manually selected features.

The use of SMOTETomek as an over- and undersampling approach proved effective in balancing the number of patients with or without progression. By creating a dataset with the same number of patients in each class, the predictions of Gaussian NB were not influenced by the prevalence of either class; rather, each prediction relied solely on the input clinical and radiomics features. 

**Figure 4:**
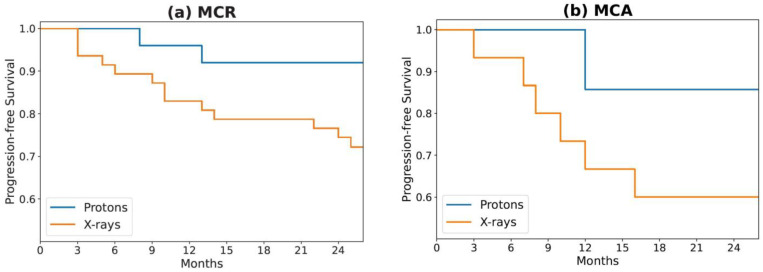
Kaplan–Meier survival curves for progression-free survival of (**a**) MCR and (**b**) MCA patients, stratified by the type of radiation received during treatment.

In both MCR and MCA cohorts, we noticed a lower numerical progression rate for the patients treated with proton therapy compared to x-ray therapy (MCR: 8% vs. 32%; MCA: 14% vs. 47%; Table 1). The ‘Radiation Type’ clinical feature was identified as the top feature from the Mann–Whitney U test. Additionally, it was detected as the best single-variable model, which suggests a strong predictive value compared to other clinical, radiomics, and principal component features. This difference cannot be explained simply by the small difference observed between patient epidemiology (see Table 1). At both institutions, the radiation dose was calculated using a relative biological effective (RBE) value of 1.1. The difference in progression (Figure 4) raises the question of the role that RBE plays in tumor control probability and whether 1.1 is an adequate value, but that inquiry is beyond the scope of this paper. Because conclusions regarding radiation type are limited by our small sample size, future studies should examine its prognostic significance in larger cohorts.

While pre- and post-treatment 18-F-fluorodeoxyglucose PET-CT is the standard for diagnosis and surveillance of head-and-neck-squamous cell cancers, others have applied different imaging modalities for radiomics-based progression prediction, including contrast-enhanced and non-contrast CT, as well as diffusion-weighted, T1, and T2 MRI sequences [14]. Future work could explore whether other imaging modalities or different PET tracers, such as fibroblast activation protein inhibitor (FAPI)-PET [33,34], offer information complementary to published 18-F-fluorodeoxyglucose PET-based models.

The presented study may be limited by selection bias and other inherent biases associated with any retrospective analysis. Additionally, the small sample size in the cohorts constitutes a limitation for the training and validation of the models. We should note that the two institutions, MCR and MCA, may conduct similar practices that could result in homogeneous treatment outcomes across the institutions. Future work should focus on larger cohorts and additional independent cross-validation to enhance the generalizability of the presented model to multi-institutional and multi-scanner datasets.

## 5. Conclusions

The radiomics-based classifiers built in this work used a multi-scanner dataset to predict disease progression of HPV-positive OPC patients from an external institution. Our results aid in the identification of pre-treatment clinical and radiomics features that provide risk-stratifying information for post-treatment progression. These features should be further evaluated for their ability to inform individualized treatment and follow-up strategies tailored to individual patients.

## Figures and Tables

**Table 1:** Patient clinical and pathological data.

		MCR		MCA	
Radiation Type		X-rays	Protons	X-rays	Protons
Total Patients		47	25	15	7
Any Progression					
	Yes	15	2	7	1
	No	32	23	8	6
Age at Diagnosis (y)					
	Mean	62.5	64.9	63.2	65.8
	Range	48.1–77.7	42.3–81.0	46.4–86.9	55.1–76.3
T Category					
	T0	0	0	0	0
	T1	5	0	0	0
	T2	15	3	4	5
	T3	8	9	4	1
	T4	19	13	7	1
N Category					
	N0	6	1	0	1
	N1	1	7	4	1
	N2	35	17	9	5
	N3	5	0	2	0
Concurrent Chemotherapy					
	Yes	45	24	15	7
	No	2	1	0	0

MCR: Mayo Clinic, Rochester, Minnesota. MCA: Mayo Clinic, Phoenix, Arizona.

**Table 2:** Features selected via forward feature selection scheme. Models of incremental number of features were selected by adding one feature at a time and retaining the variable that resulted in the largest cross-validation f1 score.

	Dimensionality Reduction Method	
No. of Input Features	Manually Filtered Set	Machine-Driven Set
1	Radiation Type	Radiation Type
2	Radiation Type, ROIs_MajorAxisLength	Radiation Type, PCA 3
3	Radiation Type, ROIs_MajorAxisLength, LargestROI_Flatness	Radiation Type, PCA 3, PCA 6
4	Radiation Type, ROIs_MajorAxisLength, LargestROI_Flatness, ROIs_Flatness	Radiation Type, PCA 3, PCA 6, PCA 4
5	Radiation Type, ROIs_MajorAxisLength, LargestROI_Flatness, ROIs_Flatness, LargestROI_Skewness	Radiation Type, PCA 3, PCA 6, PCA 4, Primary Stage N
6	Radiation Type, ROIs_MajorAxisLength, LargestROI_Flatness, ROIs_Flatness, LargestROI_Skewness, LargestROI_SurfaceArea	Radiation Type, PCA 3, PCA 6, PCA 4, Primary Stage N, PCA 5

PCA: Principal component analysis. Nomenclature for radiomics features: VolumeOfInterest_FeatureName.

## Data Availability

Data supporting the reported results can be obtained from the corresponding author.
